# Human Umbilical Cord Matrix Mesenchymal Stem Cells Suppress the Growth of Breast Cancer by Expression of Tumor Suppressor Genes

**DOI:** 10.1371/journal.pone.0123756

**Published:** 2015-05-05

**Authors:** Naomi Ohta, Susumu Ishiguro, Atsushi Kawabata, Deepthi Uppalapati, Marla Pyle, Deryl Troyer, Supriyo De, Yongqing Zhang, Kevin G. Becker, Masaaki Tamura

**Affiliations:** 1 Department of Anatomy and Physiology, College of Veterinary Medicine, Kansas State University, Manhattan, 66506, United States of America; 2 Gene Expression and Genomics Unit, NIH Biomedical Research Center, National Institute on Aging, NIH, Baltimore, MD, 21224, United States of America; University of Alabama at Birmingham, UNITED STATES

## Abstract

Human and rat umbilical cord matrix mesenchymal stem cells (UCMSC) possess the ability to control the growth of breast carcinoma cells. Comparative analyses of two types of UCMSC suggest that rat UCMSC-dependent growth regulation is significantly stronger than that of human UCMSC. Their different tumoricidal abilities were clarified by analyzing gene expression profiles in the two types of UCMSC. Microarray analysis revealed differential gene expression between untreated naïve UCMSC and those co-cultured with species-matched breast carcinoma cells. The analyses screened 17 differentially expressed genes that are commonly detected in both human and rat UCMSC. The comparison between the two sets of gene expression profiles identified two tumor suppressor genes, adipose-differentiation related protein (ADRP) and follistatin (FST), that were specifically up-regulated in rat UCMSC, but down-regulated in human UCMSC when they were co-cultured with the corresponding species’ breast carcinoma cells. Over-expression of FST, but not ADRP, in human UCMSC enhanced their ability to suppress the growth of MDA-231 cells. The growth of MDA-231 cells was also significantly lower when they were cultured in medium conditioned with FST, but not ADRP over-expressing human UCMSC. In the breast carcinoma lung metastasis model generated with MDA-231 cells, systemic treatment with FST-over-expressing human UCMSC significantly attenuated the tumor burden. These results suggest that FST may play an important role in exhibiting stronger tumoricidal ability in rat UCMSC than human UCMSC and also implies that human UCMSC can be transformed into stronger tumoricidal cells by enhancing tumor suppressor gene expression.

## Introduction

Umbilical cord matrix mesenchymal stem cells (UCMSC) are derived from the gelatinous connective tissue of the umbilical cord, Wharton’s jelly. UCMSC exhibit primitive stem cell characteristics which include self-renewability and multipotency. They express similar stem cell markers with those expressed in bone marrow derived mesenchymal stem cells [1]. UCMSC can differentiate into cardiomyocytes, neuron-like cells, osteocytes, endothelial cells, and pancreatic islet-like cell clusters [2–4].

Mesenchymal stem cells including UCMSC home to inflammatory regions including cancers, which makes them useful as virus or nanoparticle-loaded gene or drug carriers [5,6]. Recent findings show that naïve human or rat UCMSC suppress the growth of several kinds of tumors [7–9]. Rat naïve UCMSC completely abolished the growth of rat mammary tumors without recurrence for 100 days [7]. The growth of pancreatic and lung cancer xenografts were also significantly suppressed by rat UCMSC therapy in immunocompetent mice [8,9]. The *in vitro* studies showed a decrease in breast cancer cell growth by indirect co-culture of naïve UCMSC and breast cancer cells [10]. Conditioned medium with naïve UCMSC also suppressed the growth of breast, lung, and pancreatic cancer cells [8–10]. Although the mechanisms by which naïve UCMSC suppress the tumor growth is not fully elucidated, a few potential mechanisms have been proposed; UCMSC produce transmissive factors and cause cell cycle arrest and apoptosis in tumor cells; they activate anti-tumor immune responses in cancer-bearing animals [9–13]. It is also suggested that naïve UCMSC communicate with adjacent cancer cells by exchanging chemical signals with each other: this communication is most likely mediated by cytokines and growth factors. However, this cytokine or growth factor-mediated communication is not fully clarified.

On the other hand, although both human and rat naïve UCMSC can suppress tumor growth, the tumor growth inhibition by human UCMSC is not as strong as that of rat UCMSC. In [^3^H]-Thymidine uptake assay, a small number of rat UCMSC (1:15) suppressed the growth of rat breast carcinoma cells more than 90%, whereas a much higher number of human UCMSC (1:2) suppressed only 50% of the growth of human breast cancer cells [7,10]. This difference in cell growth inhibition may suggest that the two types of UCMSC exhibit fundamental differences in cell-to-cell communication by cytokines and growth factors.

Accordingly, the present study was conducted to discover the key mechanisms by which rat and human UCMSC attenuate tumor growth by defining UCMSC-produced cytokines and growth factors. To conduct this study, we hypothesized that; 1) human and rat UMCSC express genes differently when they co-exist with breast carcinoma cells; 2) tumoricidal activities of human and rat UCMSC are dependent on differentially expressed genes and their products; 3) expression manipulation of identified rat UCMSC tumoricidal genes in human UCMSC will generate human UCMSC armed with enhanced tumoricidal ability. Proving these hypotheses may represent the molecular mechanism by which naïve UCMSC inhibit tumor growth. Furthermore, this principle can be applied to generate strongly tumoricidal human UCMSC for breast cancer treatment.

## Materials and Methods

### Cell culture

Human and rat UCMSC were prepared from Wharton’s jelly, which is the matrix of the umbilical cord, as previously described [1,2,7,14]. Practically, the cords were collected by hospital’s medical personnel under the supervision of the gynecologist and were given with no donor-identifying information. The informed consent procedure was handled by hospital staff so that the donors were informed of the potential use of their umbilical cords for medical research [2]. Additionally, we were assured that the cords were free from known infectious diseases. The human biological samples were sourced ethically and their research use was approved by the Kansas State University Institutional Review Board (Protocol # 3515). UCMSC were maintained in a defined medium, containing a mixture of 56% low glucose Dulbecco’s Modified Eagle Medium (DMEM; Life Technologies, Grand Island, NY), 37% MCDB 201 (Sigma, St. Louis, MO), 2% fetal bovine serum (FBS) (Atlanta Biologicals, Lawrenceville, GA), 1x insulin-transferrin-selenium-X (Life Technologies), 1x ALBUMax I (Life Technologies), 1x penicillin/streptomycin (Life Technologies), 10nM dexamethasone, 100μM ascorbic acid 2-phosphate, 10ng/ml epidermal growth factor, and 10ng/ml platelet derived growth factor-bb (all from Sigma, St. Louis, MO). MDA-231 human mammary adenocarcinoma cell line, kindly provided by Dr. F. Marini (M.D. Anderson Cancer Center, TX), were maintained in Minimum Essential Media (MEM) alpha medium (Life Technologies) supplemented with 10% FBS, 1x penicillin/streptomycin, 1% non-essential amino acids, and 1% L-glutamine. The Mat B III rat mammary adenocarcinoma cell line was purchased from American Type Culture Collection (Manassas, VA). The Mat B III cells were maintained in McCoy's 5A modified medium (Life technologies) supplemented with 10% FBS and 1x penicillin/streptomycin. All of the cell lines were maintained at 37°C in a humidified atmosphere containing 5% CO_2_.

### Co-culture and RNA isolation

An indirect cell culture using Transwell culture dishes (Corning Inc., Lowell, MA) was performed to collect RNA samples for microarray study. In brief, human (5x10^4^ cells) or rat (1x10^5^ cells) UCMSC were seeded in defined medium in the bottom of a 6-well or 10cm Transwell culture dish, respectively. After allowing UCMSC to settle for 24 hours at 37°C in a humidified incubator at 5% CO_2_, MDA-231 cells (5x10^5^ cells) or Mat B III cells (1.5x10^6^ cells) were added in Transwell inserts (0.4μm pore size). Naïve UCMSC were cultured in identical conditions as the co-cultured UCMSC without carcinoma cell addition to the inserts. After 48 hours at 37°C, total RNA was extracted from naïve UCMSC or UCMSC co-cultured with carcinoma cells using TRIzol following the manufacturer’s instruction (Life Technologies). Concentration and quality of total RNA were determined by the NanoDrop ND-8000 spectrophotometer (NanoDrop Technologies, Inc., Wilmington, DE) and Agilent 2100 bioanalyzer (Agilent Technologies, Inc. Santa Clara, CA), respectively.

### Microarray and data analysis

Microarray experiments including RNA quality evaluation, hybridization, and initial data analysis were carried out at the National Institute on Aging, National Institutes of Health (Baltimore, MD) according to previously described methods [15]. The data are held in the Gene Expression Omnibus (GEO) database under accession #GSE64839. Detailed methods for microarray experiments have been described elsewhere [12]. The microarray dataset was analyzed with DIANE 6.0, a spreadsheet-based microarray analysis program, as described previously [12,15]. The DIANE microarray analysis program can be found online at http://www.grc.nia.nih.gov/branches/rrb/dna/diane_software.pdf.

### Quantitative real-time PCR (qRT-PCR)

The expression of adipose-differentiation related protein (ADRP) and follistatin (FST) in microarray analysis were reevaluated by qRT-PCR using the same RNA samples as those used for the microarray. qRT-PCR was carried out using the iScript One-Step RT-PCR Kit with SYBR Green (Bio-Rad, Hercules, CA), and the reactions were conducted on the StepOnePlus Real-Time PCR System (Applied Biosystems). The qRT-PCR was performed as follows: a 10 min initial incubation at 50°C was followed by 45 cycles of 10 seconds at 95°C, 20 seconds at 58°C, and 50 seconds at 72°C, followed by final extension at 72°C for 4 min. The results were quantified by the comparative Ct method [16]. The sequences of primers used are described in Table 1.

**Table 1:** Primers used for qRT-PCR.

Species	Genes	Forward (5’-3’)	Reverse (5’-3’)	References
Human	*ADRP*	CTCATGGGTAGAGTGGAA-AAGGAGCATTGG	TTGGATGTTGGACAGGAG-GGTGTGGCACGT	[17]
	*FST*	TGTGCCCTGACAGTAAGTCG	GTCTTCCGAAATGGAGTTGC	This study
Rat	*Adrp*	CATTCAAGACCAGGCCAAAC	AGGAGGTAACATTGCGGAAC	This study
	*Fst*	TGCTGCTACTCTGCCAATTC	TGCAACACTCTTCCTTGCTC	This study

### Adenovirus construction and transduction

Green fluorescent protein(GFP)-tagged adenoviral vectors for human ADRP (Ad-ADRP) and FST (Ad-FST) genes were constructed using human ADRP and FST cDNA plasmids from Open Biosystems (Huntsville, AL) by Vector Biolabs (Philadelphia, NJ). Adenovirus encoding β-galactosidase (Ad-LacZ) gene was prepared as described previously [18]. Adenoviral vectors were expanded and titred by following the BD Adeno-X Expression system manual (Clontech Laboratories, Inc, Mountain View, CA). The gene transduction to human UCMSC was carried out by following the procedure described in the previous study [19]. Transduction efficiencies of Ad-ADRP and Ad-FST to the human UCMSC were determined by qRT-PCR as described above. GFP expression of gene transduced UCMSC was observed under a fluorescent microscope.

### Evaluation of the effect of ADRP or FST over-expressing human UCMSC on the growth of MDA-231 cells in co-culture

The effect of ADRP or FST over-expressing human UCMSC on the growth of MDA-231 cells was evaluated by direct co-culture and 3-(4,5-Dimethylthiazol-2-yl)-2,5-diphenyltetrazolium bromide (MTT) assay. Human UCMSC transduced with Ad-ADRP, Ad-FST, or Ad-LacZ in 50 multiplicity of infection (MOI) were seeded (1,250 cells/well) in a 24-well plate and incubated at 37°C in a humidified, 5% CO_2_ incubator allowed to attach to the plate. Twenty-four hours later, MDA-231 cells (12,500 cells/well) were seeded directly into the wells containing gene transduced human UCMSC and co-cultured for 48 hours at 37°C. The total cell proliferation of MDA-231 cells and co-cultured human UCMSC was quantified by MTT assay as described previously [18].

### Evaluation of the effect of conditioned medium obtained from engineered human UCMSC on the growth of human breast carcinoma cells

The effect of conditioned media collected from human UCMSC transduced with ADRP or FST gene on the growth of MDA-231 cells was evaluated by conventional cell culture and MTT assay. Un-conditioned defined medium was used as control. In brief, Ad-ADRP,-FST or-LacZ (50 MOI) were transduced on human UCMSC. The media conditioned with various engineered human UCMSC were collected after 48 hours of incubation and diluted with defined medium in a 1:1 ratio. MDA-231 cells seeded in 96 well plates (2000 cells/well) were incubated for 24 hours, and then cultured for 48 hours in various conditioned media. Cell proliferation of MDA-231 cells was quantified by MTT assay as described previously [18].

### Evaluation of the effect of ADRP or FST over-expression in human breast carcinoma cells on formation and growth of colonies

Two layer-colony forming assay was carried out as a three dimensional colony growth of human breast carcinoma cells by following the method described previously [10]. Briefly, 0.5 ml of 0.9% agar (Sea Plaque agarose, Cambrex Bio Science Rockland, Inc. Rockland, ME) in MEM α medium supplemented with 10% FBS was poured into the wells of a 12-well tissue culture plate as a bottom layer. After solidification, 25,000 MDA-231 cells transduced with 50 MOI Ad-ADRP,-FST or-LacZ were suspended in 0.5ml 0.5% agarose in MEM α medium containing 10% FBS and layered over the bottom layer. The cells were incubated at 37°C with 5% CO_2_ for growth of colonies. On days 7 and 12, colony growth was evaluated by an automated phase contrast microscope equipped with Micro Analysis Suite (Olympus CKX41, Center Valley, PA). Colonies with an area greater than 5,000 μm^2^ were counted using Micro Suite Five software. The ratio of colony growth of MDA-231 cells was determined between days 7 and 12.

### Evaluation of the effect of FST over-expressing human UCMSC on human breast carcinoma cell metastasis in mouse lungs

All animal experiments were carried out under strict adherence with the Kansas State University Institutional Animal Care and Use Committee (IACUC) protocol. The protocol was approved by the Kansas State University IACUC (Protocol # 3015) and Institutional Biosafety Committee. All mice were sacrificed under isofluorane anesthesia and all efforts were made to minimize suffering. Sixteen severe combined immunodeficiency (CB17/SCID) mice obtained from Charles River (Wilmington, MA) were housed in an animal care facility and held for 10 days to acclimatize. Each mouse was injected with 2x10^6^ MDA-231 cells through the tail vein. Six days after cancer cell inoculation, mice were randomly divided into three treatment groups; (1) phosphate buffered saline (PBS; n = 5) as a no-treatment control; (2) FST over-expressing human UCMSC (n = 6); or (3) LacZ over-expressing human UCMSC (n = 5) as a control for adeno-virus transduced UCMSC. On days 6, 13, and 20 after the MDA-231 cell inoculation, mice were injected intravenously with 200μl PBS, FST or LacZ over-expressing human UCMSC (5x10^5^ cells/mouse) in 200μl PBS. The mouse body weights were monitored every other day. Four weeks after MDA-231 cell transplantation, all mice were sacrificed and major organs including brains, lungs, livers, kidneys, and mesenteries were examined for their potential tumor growth. Numbers of tumor nodules in the lungs were counted under stereomicroscope. The *in-vivo* experiment was conducted one time.

### Histological analysis of cell proliferation and apoptosis in metastatic lung tumors

Lungs were fixed in 10% buffered-formalin, paraffin embedded and sectioned at 4μm for histological analysis. After deparaffinization of sections, heat-induced epitope unmasking was performed in citrate buffer followed by incubation with 3% H_2_O_2_/methanol for 5 minutes to block endogenous peroxidase activity. To analyze cell proliferation in tumors, sections were immunostained with Ki-67 (1:100; Catalog #: ab15580, Abcam, Cambridge, MA) for 1 hour at 37°C then treated with a biotin-conjugated anti-rabbit IgG antibody (1:100; Catalog #: BA-1000, Vector Laboratories, Burlingham, CA) for 1 hour at 37°C. Sections were then treated with the avidin-biotin-peroxidase complex reagent (Vector Laboratories, Burlingham, CA) for 40 minutes at 37°C. Reactions were developed with 3,3'-diaminobenzodine tetrahydrochloride (Sigma, St. Louis, MO) and counterstained lightly with Mayer`s hematoxylin. To determine apoptosis in tumors, a terminal deoxynucleotidyl transferase—mediated dUTP nick end labeling (TUNEL) assay was conducted using the DeadEnd Colorimetric TUNEL System (Catalog #: G7130, Promega, Madison, WI), according to the manufacturer's instructions with a slight modification [20]. The percentage of Ki-67 positive cells area and the absolute number of TUNEL positive cells in the 5 randomly selected tumor area were measured for the cell proliferation index and apoptotic index, respectively, by ImageJ software.

### Statistical analysis

All values are expressed as means ± standard error (S.E.) for all *in vitro* and *in vivo* experiments. Statistical significance was assessed by one-way ANOVA following Tukey's test or t-test. Statistical significance was set at *p* < 0.05.

## Results

### Differential gene expression in human and rat UCMSC by microarray analysis

**Fig 1:**
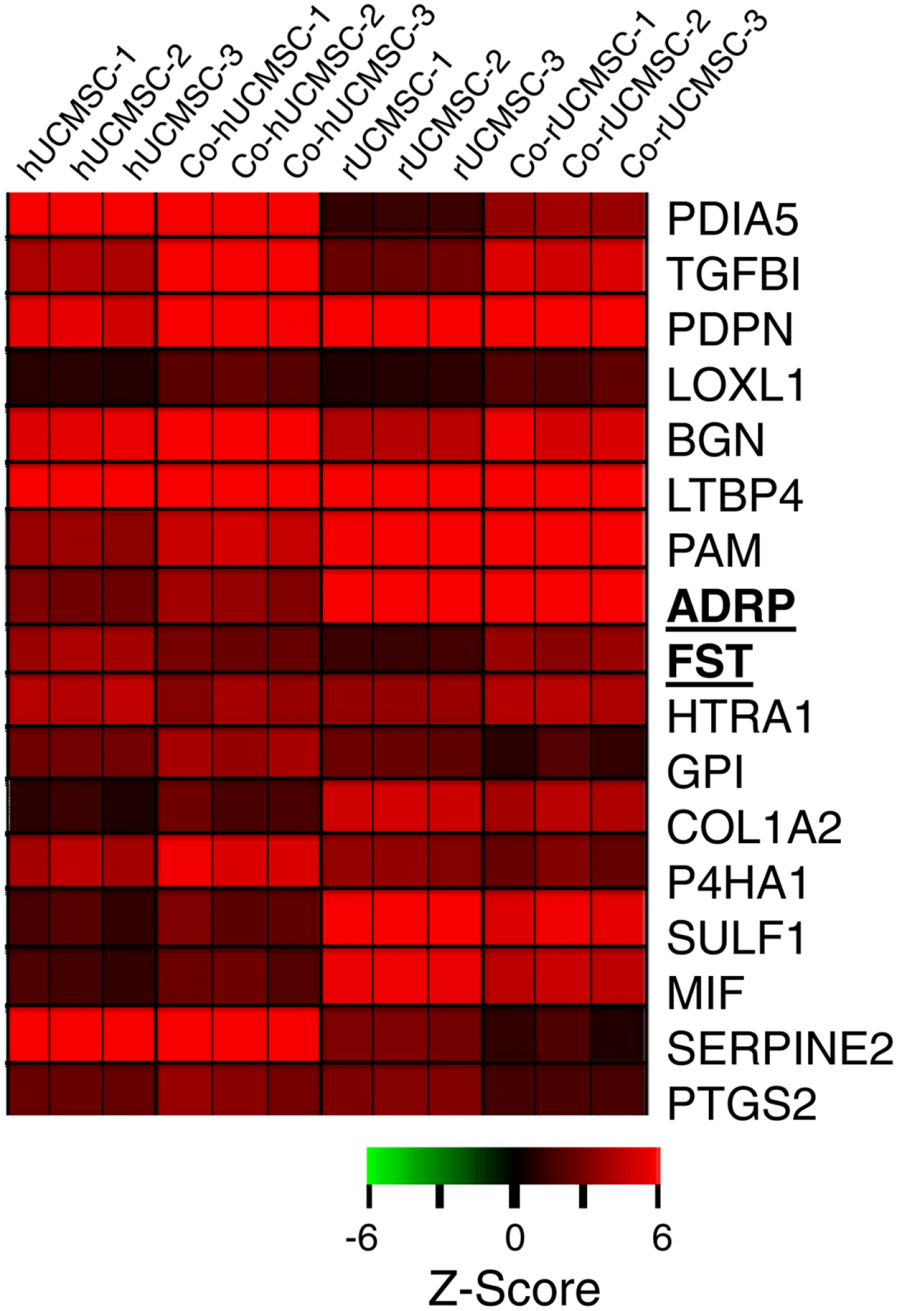
Clustering analysis of significant genes in rat UCMSC (rUCMSC) and human UCMSC (hUCMSC) co-cultured with mammary carcinoma cells of their respective species; Mat B III rat breast carcinoma cells or MDA-231 human breast carcinoma cells. The genes differentially expressed between naïve human or rat UCMSC and those co-cultured with mammary carcinoma cells are listed on the row and the samples are shown on the column. Red are higher expression and black to green are lower expression. This analysis suggests that a short time co-culture with mammary carcinoma cells significantly modifies expression of multiple tumor-suppressor and-promoter genes in both human and rat UCMSC.

**Fig 2:**
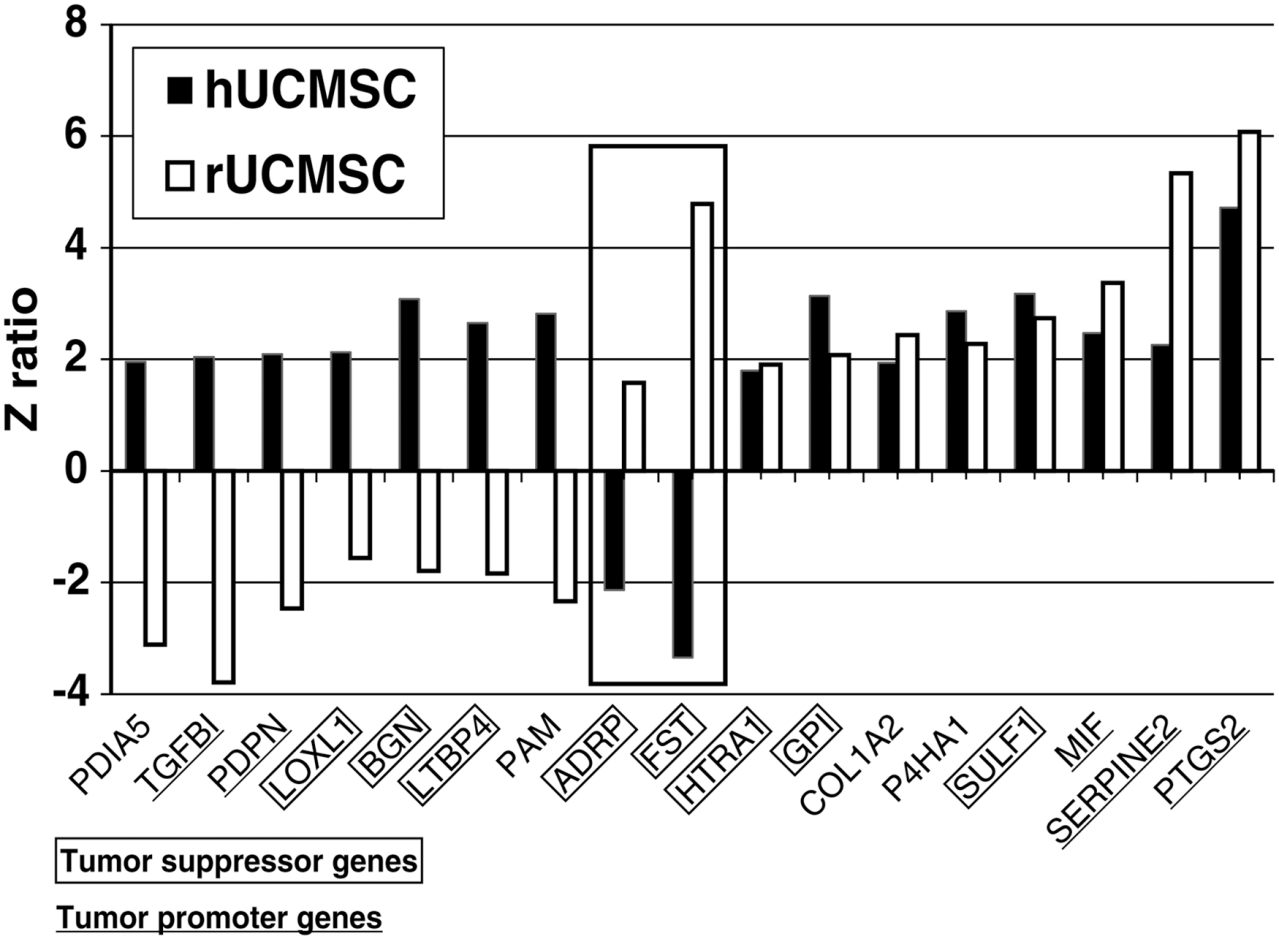
Z ratios of the 17 commonly expressed genes in both hUCMSC and rUCMSC co-cultured with breast carcinoma cells. The solid bars indicate gene expression in hUCMSC and the white bars indicate gene expression in rUCMSC.

The characteristics of human and rat UCMSC are described previously [2,7]. Differentially expressed genes in human or rat naïve UCMSC and those co-cultured with corresponding species’ mammary carcinoma cells were determined by genome-wide microarray analysis as described in the Method section (Accession #GSE64839). Uniquely expressed genes were further screened by the following three criteria; 1) gene expression difference should be at least 1.5 fold different between naïve UCMSC and those co-cultured with breast carcinoma cells; 2) the genes must encode secretory proteins; and 3) the genes are associated with cell growth regulation. By the analysis of z-normalization of the hybridization signals, 43 genes were selected in human UCMSC and 64 genes were selected from rat UCMSC. By comparing these selected genes between human and rat UCMSC, 17 genes were discovered to be commonly expressed in both UCMSC (Fig 1). Eight of these genes were up-regulated in both human and rat UCMSC. Two genes were up-regulated in rat UCMSC but down-regulated in human UCMSC, while the other seven genes were up-regulated in human UCMSC but down-regulated in rat UCMSC (Fig 2). Furthermore, eight genes were reported as tumor suppressor genes and five genes were reported as tumor promoter genes while the other four genes are related to cell growth but their functions in tumor growth are unknown (Table 2). Two tumor suppressor genes, ADRP and FST, which were up-regulated in rat UCMSC but down-regulated in human UCMSC were further characterized as potential candidate genes to exhibit stronger tumoricidal activity in rat UCMSC than in human UMCSC. The mRNA expressions of ADRP and FST in human and rat UCMSC were further verified by qRT-PCR. Both ADRP and FST mRNA expressions were consistently up-regulated in rat UCMSC co-cultured with Mat B III cells (8.5 ± 0.3 and 1.8 ± 0.05 folds, respectively). In human UCMSC co-cultured with MDA-231 cells, although FST mRNA expression was down-regulated (0.4 ± 0.1 fold) as observed in the microarray study, ADRP expression was up-regulated (2.7 ± 0.3 fold).

**Table 2:** Function and expression levels of differentially expressed genes in human and rat UCMSC.

Tumor promoter genes^1^	Rat	Human	Ref.	Tumor suppressor genes^2^	Rat	Human	Ref.
PTGS2 (COX2)	+	+	[30]	SULF1	+	+	[12,31]
SERPINE2	+	+	[32,33]	GPI	+	+	[12]
MIF	+	+	[29]	HTRA1	+	+	[34]
PDPN	-	+	[35]	ADRP	+	-	[12,27]
TGFBI	-	+	[36]	FST	+	-	[21]
**Function unknown genes** ^**3**^	**Rat**	**Human**		LTBP4	+	-	[37,38]
P4HA1, COL1A2,	+	+		BGN	+	-	[39]
PAM, PDIA5	-	+		LOXL1	+	-	[40]

Positive (+) indicates upregulation and negative (-) indicates down regulation.

^1^ PTGS2 (COX2), prostaglandin-endoperoxide synthase 2, SERPINE2, serpin peptidase inhibitor clade E, MIF, macrophage migration inhibitory factor, PDPN, podoplanin, TGFBI, transforming growth factor, beta-induced, 68kDa.

^2^ SULF1, sulfatase 1, GPI, glucose-6-phosphate isomerase, HTRA1, HtrA serine peptidase 1, ADRP, adipose-differentiation related protein, FST, follistatin, LTBP4, latent transforming growth factor beta binding protein 4, BGN, biglycan, LOXL1, lysyl oxidase-like 1.

^3^ P4HA1, prolyl 4-hydroxylase, alpha polypeptide I, COL1A2, collagen, type 1, alpha2, PAM, peptidylglycine alpha-amidating monooxygenase, PDIA5, protein disulfide isomerase family A, member 5.

### Human UCMSC over-expressing FST inhibited the growth of MDA-231 cells *in vitro*

To evaluate the effects of ADRP or FST over-expression in human UCMSC on breast carcinoma cell growth, adenoviral vectors encoding ADRP (Ad-ADRP) and FST (Ad-FST) were individually constructed using GFP encoding expression vector. Gene transduction by Ad-ADRP or Ad-FST was confirmed by observing GFP co-expression in the cells. A good even expression of GFP was detected in human UCMSC under a fluorescent microscope when they were transduced with 50 MOI vectors.

**Fig 3:**
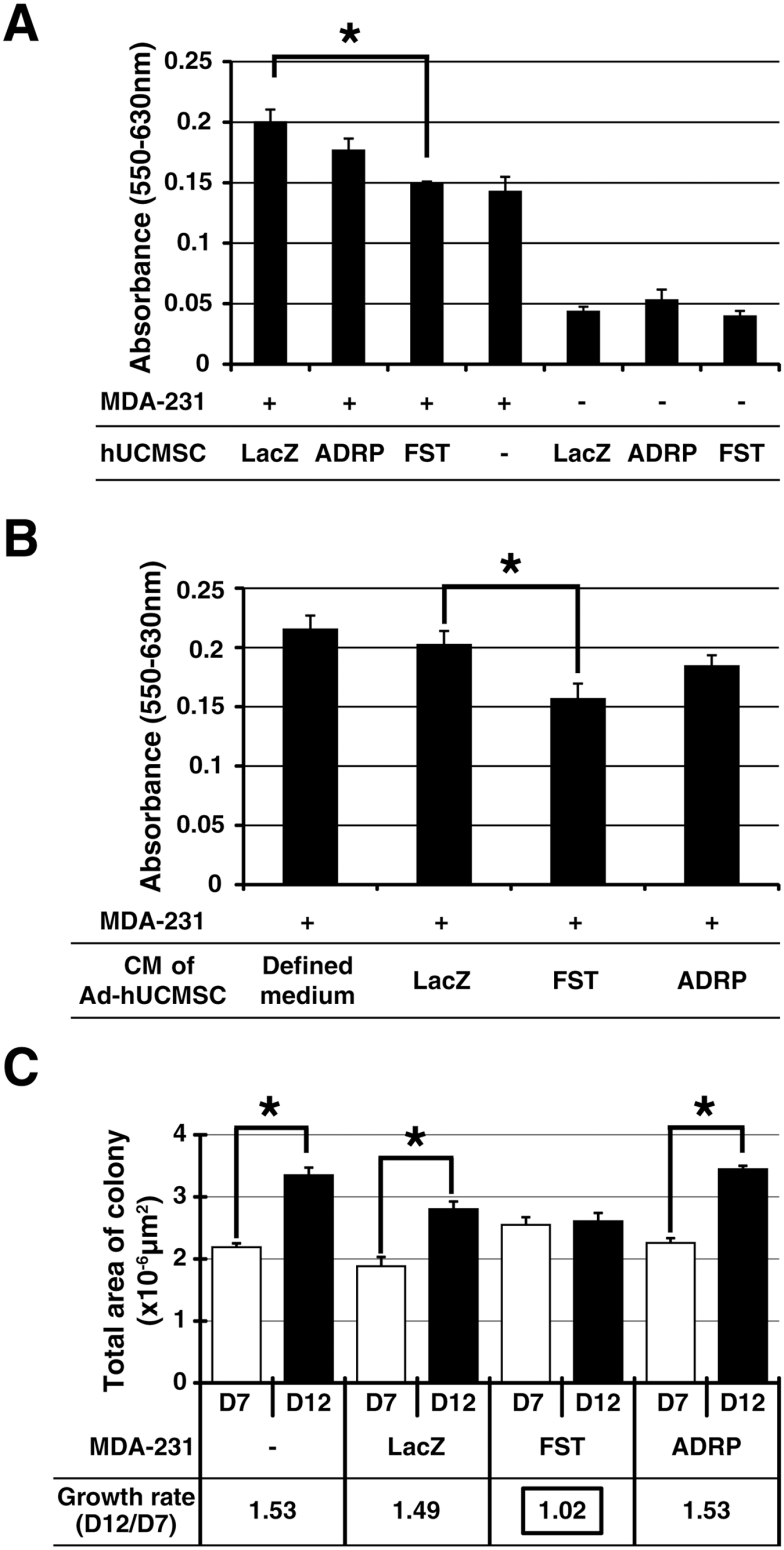
The growth of MDA-231 cells was significantly attenuated by co-culture with FST-hUCMSC (A), the conditioned medium from FST-hUCMSC (B), or by direct over-expression of FST (C). A, MDA-231 cells were co-cultured with either LacZ-, ADRP- or FST-hUCMSC for 48 hours. Cell growth was measured by MTT assay (panels A and B). Transduction of hUCMSC with adenoviral vector did not affect the cell growth. FST-hUCMSC, but not ADRP-hUCMSC significantly attenuated the growth of co-cultured carcinoma cells to the same level of MDA-231 cells cultured alone. B, the growth of MDA-231 cells was significantly attenuated by culturing with the conditioned medium (CM) from Ad-FST but not-LacZ or-ADRP transduced human UCMSC or defined medium (DM). C, the growth of MDA-231 cells was significantly attenuated by the direct transduction of Ad-FST but not by-LacZ or-ADRP. The colony growth rate of the FST-transduced MDA-231 cells from Day 7 to 12 was 1.02 (no growth), while the other gene-transduced cells colonies grew approximately 1.5 times bigger on Day 12 compared to Day 7. * *p* < 0.05.

The effect of Ad-FST transduced human UCMSC (FST-hUCMSC) on the growth of MDA-231 cells was evaluated by direct co-culture of the two types of cells in 2D cultures. As shown in Fig 3A, direct co-culture of FST, but not ADRP, transduced human UCMSC (ADRP-hUCMSC) with MDA-231 cells (1:10 ratio) decreased the overall cell growth significantly. Furthermore, to evaluate indirect effect of the gene transduced human UCMSC on the growth of MDA-231 cells, MDA-231 cells were cultured with the conditioned medium obtained from gene transduced UCMSC and the growth of MDA-231 cells was evaluated by MTT assay (Fig 3B). The growth of MDA-231 cells was significantly attenuated when cultured with the conditioned medium from FST-hUCMSC as compared to that cultured in LacZ transduced human UCMSC (LacZ-hUCMSC). However, the conditioned medium from ADRP-hUCMSC showed only a slight decrease in cell growth. The experiment utilizing the conditioned medium supports the idea that FST appears to be secreted in the culture medium and UCMSC dependent cell growth attenuation is at least in part mediated through proteins secreted from the UCMSC.

In the 3D co-culture in which MDA-231 cells were cultured in 0.4% soft agar gel, the colony size and numbers of FST transduced, but not LacZ, or ADRP transduced MDA231 cells were significantly inhibited 12 days after gene transduction (Fig 3C). These results suggest that the FST expression in MDA-231 cells also inhibits cell growth in autocrine and paracrine manners. On the other hand, the inhibitory effect of the ADRP gene on tumor growth was negligible in the present study (Fig 3). This very weak tumor growth regulation of ADRP may be associated with ADRP-dependent cell differentiation of UCMSC. In support of this idea, a significant lipid accumulation was observed when human UCMSC were transduced with ADRP (S1 Fig).

### Human UCMSC transduced with FST inhibited growth of MDA-231 xenografts in mice

**Fig 4:**
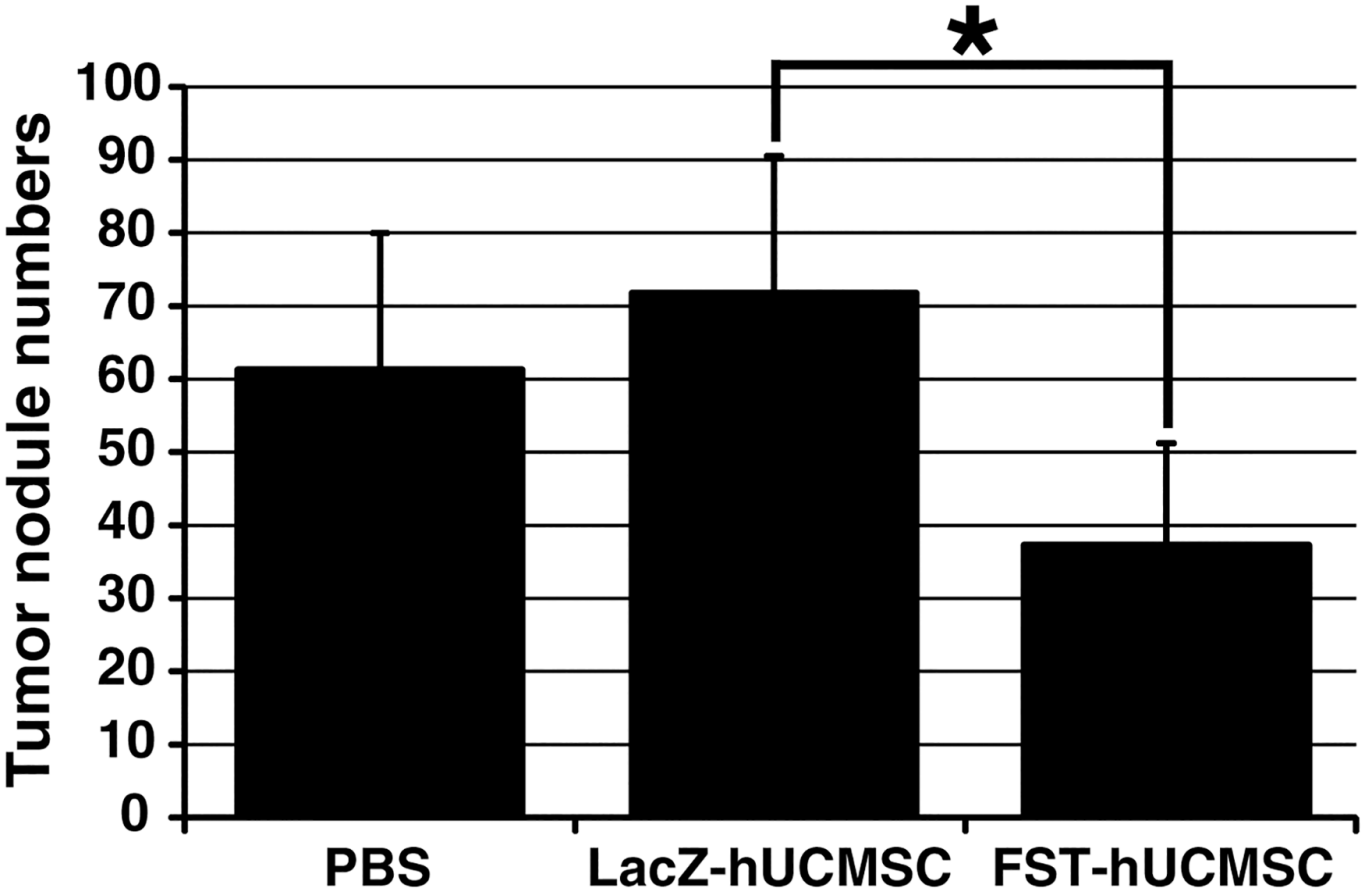
Treatment with FST-hUCMSC significantly attenuated development of metastatic tumor in the mouse lung. The numbers of tumor nodules in the mouse lung were counted under a dissection microscope 30 days after the inoculation of the MDA-231 cells. The bar graph represents the average number of tumor nodules in each treatment group. * *p* < 0.05 compared to LacZ-hUCMSC treated mice.

To further evaluate the significance of the FST gene in tumor growth inhibition, FST-hUCMSC was tested to determine whether FST-hUCMSC attenuated the growth of MDA-231 grafts in a breast cancer lung metastasis model. For this *in vivo* mouse study, a lung metastasis model of MDA-231 breast carcinoma cells was used since the lung is one of the most prominent sites of breast cancer metastasis and this model is reproducible. Three weekly treatments with half a million FST-hUCMSC significantly attenuated metastatic tumor growth in the lung; the tumor nodule number in the FST-hUCMSC treated mouse lungs obtained one week after the third treatment was significantly smaller than those in LacZ-hUCMSC or PBS treated mouse lungs (Fig 4). No tumors were observed in other organs including brain, liver, kidney, and mesentery. These results clearly suggest that FST expression in human UCMSC play a critically important role in suppressing tumor growth in mice.

### Histological analysis of cell proliferation and apoptosis in metastatic lung tumors

**Fig 5:**
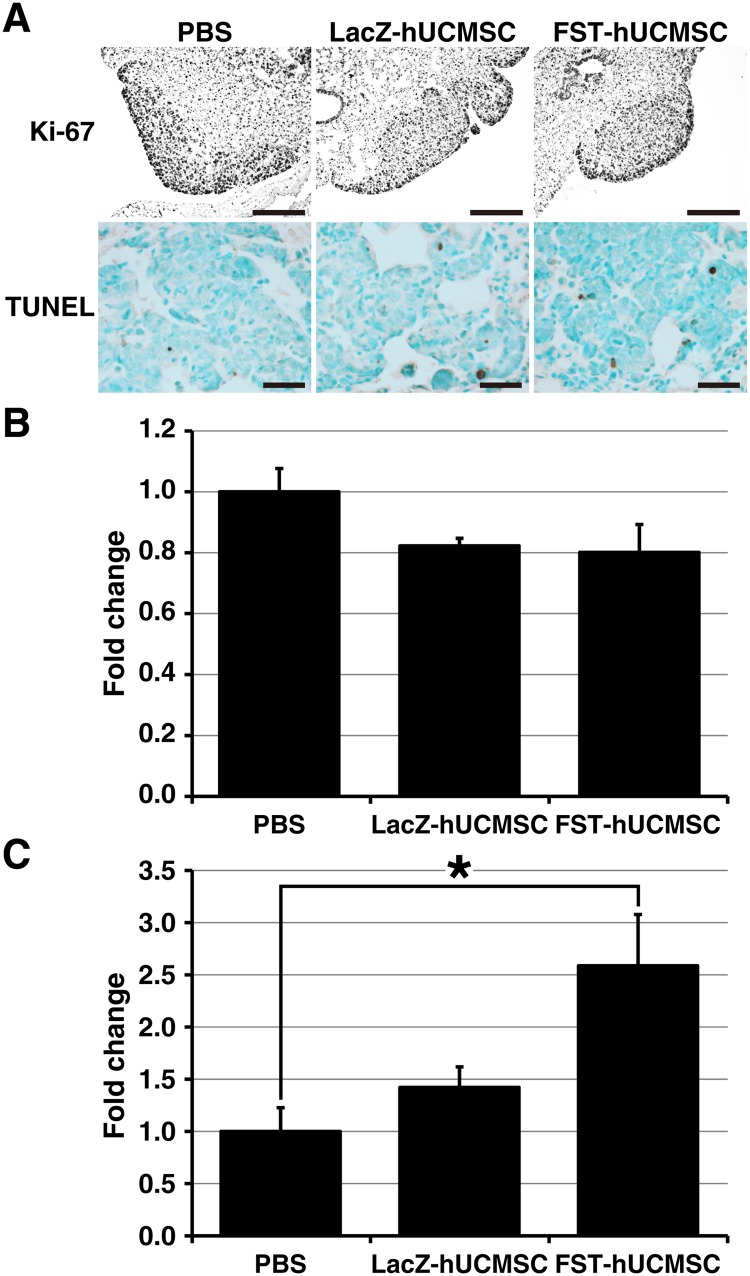
Immunohistochemical analysis of cell proliferation (A and B) and apoptosis (A and C) in MDA-231 graft tumors in SCID mouse lungs treated with either PBS, LacZ- or FST-hUCMSC. A, microscopic images of immunohistochemistry for Ki-67 (top 3 panels) at 20x and TUNEL assay (bottom 3 panels) at 40x. B, treatment with LacZ- or FST-hUCMSC had no significant effect on proliferation of the tumor cells. C, the TUNEL positive cells were significantly increased in the tumors of mice treated with FST-hUCMSC. * *p* < 0.05 as compared to the level of the PBS-treated control.

Cell proliferation and apoptosis of the metastatic tumor cells in the lungs were analyzed histologically. Immunohistochemical analysis of Ki-67 positive cells indicated that the treatment with either the LacZ- or the FST-hUCMSC slightly decreased the proliferation of tumor cells in the lungs without statistical significance (Fig 5). Apoptotic cells detected as TUNEL positive cells were located primarily in the tumor area. The number of TUNEL positive cells increased in the two treatment groups, but only the FST-hUCMSC treated group showed a significant increase in apoptotic cell numbers as compared to the PBS-treated group (*p* < 0.05, Fig 5). These results suggest that FST- hUCMSC treatment attenuated the growth of MDA-231 metastatic lung tumors mainly by increasing apoptosis and that this growth attenuation was significantly enhanced by the over-expression of the FST gene in hUCMSC.

## Discussion

The current study discovered the changes of expression patterns in tumor suppressor genes in rat and human UCMSC when they were co-cultured with carcinoma cells. Our previous study had revealed that gene expression pattern in rat UCMSC was significantly altered when they were co-cultured with rat breast carcinoma cells [12]. However, whether similar alteration of gene expression in rat UCMSC is also observed in human UCMSC when they are co-cultured with human breast carcinoma cells is unclear. In addition, although previous investigations in the tumoricidal activities of human and rat UCMSC had suggested that rat UCMSC have a stronger tumoricidal activity than human UCMSC [7,10], the molecular mechanisms by which rat UCMSC strongly attenuate the species specific growth of breast carcinoma more than human UCMSC do were unclear. Since two types of UCMSC have significant carcinoma cell growth suppressing abilities in an indirect co-culture [7,10], it is suggested that UCMSC-dependent inhibition of breast carcinoma cell growth is partially due to secreted proteins. Consequently, we hypothesized that a comparison of gene expression profiles in human and rat UCMSC co-cultured with breast carcinoma cells may provide the identity of the key players for the differential tumoricidal abilities in two types of UCMSC. Furthermore, an identification of the key mechanisms in rat UCMSC-dependent strong tumoricidal activity can be utilized to reinforce human UCMSC-dependent tumoricidal activity to the rat UCMSC level. In the present study, two tumor suppressor genes, ADRP and FST were found to be upregulated in rat UCMSC, whereas these two genes were down regulated in human UCMSC when two types of UCMSC were co-cultured with corresponding species’ breast carcinoma cells. Therefore, expression differences of these two genes in two types of UCMSC and their biological functions may explain the differential tumoricidal activities of UCMSC.

FST, which is known as an activin-binding protein, suppress the metastasis of small cell lung carcinoma [21]. FST inhibits the bioactivity of activin A, a TGF beta family protein, which is associated with lung adenocarcinoma proliferation in human patients [22]. However, the effect of activin A on breast cancer cells is still controversial [23,24]. FST over-expressing human UCMSC significantly reduced the growth of MDA-231 cells in direct and indirect co-cultures (Fig 3) which is consistent with the previous study used different cancer models [21]. Transduction of FST with MDA-231 cells also significantly suppressed the colony growth of MDA-231 cells in the colony assay (Fig 3C). Furthermore, the metastatic tumor growth of MDA-231 cells in the lung was significantly attenuated by treatment with FST-hUCMSC in mice (Fig 4). These results clearly indicate that FST expression plays a significant role in human and rat UCMSC-dependent tumoricidal activities. Indeed, a significantly higher number of apoptotic cells were detected in the FST over-expressing human UCMSC treated group (Fig 5). These results are consistent with previous reports that FST overexpression induces apoptosis in human and mouse breast carcinoma cells [25,26]. Furthermore, the anti-tumor effect shown here may also be associated with pro-apoptotic factors produced by naïve human UCMSC [10]. As reported by Ayuzawa *et al*. [10], the attenuation of Akt and MAPK pathways might also have a role in attenuating the tumor cell proliferation when human UCMSC are co-cultured with breast carcinoma cells.

ADRP has been reported as a prognosis marker of clear cell renal cell carcinoma [27]. Patients with higher expression of ADRP in clear cell renal cell carcinoma tissues had a longer survival rate than those showing lower expression [27]. Although ADRP over-expression in rat UCMSC significantly increased their growth inhibition effect against rat mammary carcinoma cells [12], their direct or indirect tumor suppressing effect against human breast carcinoma cells was not significant (Fig 3). Although the causes of functional differences between ADRP expressions in human and rat UCMSC are unclear, ADRP-dependent cell differentiation is potentially associated with this difference in two types of UCMSC. ADRP expression is shown to be associated with pre-adipocyte differentiation in several cell types [28]. Indeed, lipid accumulation was observed when human UCMSC were transduced with ADRP (S1 Fig). Therefore, it is conceivable that the initiation of adipocyte differentiation was easily induced by ADRP over-expression in human UCMSC, which may have weakened their tumoricidal ability, whereas adipocyte differentiation was not induced in rat UCMSC by ADRP over-expression. Further clarification is required on this issue.

As we hypothesized, human and rat UCMSC showed different gene expression profiles (Figs 1 and 2). A comparison of the two gene expression profiles suggests that high expression of two putative tumor suppressor genes in rat UCMSC may be associated with strong tumoricidal activity (Fig 2). Over-expression of one such gene, FST, in human UCMSC caused a significant suppression of the growth of MDA-231 cells in both cell culture and mouse studies. Although the complete depletion of the tumors was not observed by the treatment with FST-over-expressing human UCMSC (Fig 4), this study suggests that upregulation of multiple tumor suppressor genes in UCMSC is involved in both human and rat UCMSC-dependent tumor growth suppression.

The microarray analysis also indicated an upregulation of three tumor promoter genes prostaglandin-endoperoxide synthase 2 (PTGS2), serpin peptidase inhibitor, clade E, member 2 (SERPINE2), and macrophage migration inhibitory factor (MIF) in both human and rat UCMSC when they were co-cultured with breast carcinoma cells. Among these genes, extracellular MIF has been shown to act as a pro-oncogene in breast cancer [29]. Since UCMSC derived MIF might play a role as a pro-oncogene, MIF receptor expression in MDA-231 cells and Mat B III cells was investigated. MDA-231 cells have a significant level of MIF receptor expression [29], whereas our microarray data showed that Mat B III cells co-cultured with rat UCMSC did not exhibit a significant increase of MIF receptor expression which implies Mat B III cells are less sensitive to pro-oncogene, MIF. These data suggest different tumoricidal abilities of human and rat UCMSC might also be due to different sensitivities of the different breast carcinoma cells to tumor promoter proteins produced by either UCMSC. In other words, Mat B III cells are poorly sensitive to tumor promoters due to the poor receptor expression, whereas MDA-231 cells are more sensitive due to a high receptor expression for tumor promoters. A view from the regulation of the tumor promoter genes also provided a molecular mechanism of differential tumoricidal abilities of two types of UCMSCs. These results also indicate that an investigation in different combinations of human UCMSC and cancer cells is important in order to fully understand UCMSC-dependent tumoricidal activity. The present study warrants the importance of additional studies to clarify such an issue.

In conclusion, the intrinsic tumoricidal ability of UCMSC is a very unique attribute. The present study indicates that human and rat UCMSC-dependent carcinoma cell growth suppression is due at least in part to the expression modulation of multiple tumor suppressor and promoter genes. This is the first study to describe potential use of human UCMSC engineered to express an endogenous tumor suppressor gene for breast cancer treatment. This study clearly indicates that engineering human UCMSC by endogenous tumor suppressor genes can re-enforce UCMSC-dependent tumoricidal ability. It is apparent that generation of more effective human UCMSC requires further studies for efficient cell preparation and long-lasting gene transduction methods.

## Supporting Information

S1 Fig Transduction of Ad-ADRP into hUCMSC induced lipid accumulation in the cytoplasm of the cells. Naïve human UCMSC (a) were transduced by 100 MOI Ad-LacZ (b) 50 MOI (c) and 100 MOI (d) Ad-ADRP. Seven days after transduction, cells were stained by Sudan-black. Human UCMSC transduced with Ad-LacZ showed a negligible amount of oil droplets. In contrast, Ad-ADRP transduction induced a large amount of lipid accumulation in human UCMSC.
(TIFF)
