# Supplementary figures and images for: Human Umbilical Cord Matrix Mesenchymal Stem Cells Suppress the Growth of Breast Cancer by Expression of Tumor Suppressor Genes
Source: PLoS One. 2015 May 5;10(5):e0123756. doi: 10.1371/journal.pone.0123756 (PMC4420498; doi:10.1371/journal.pone.0123756)

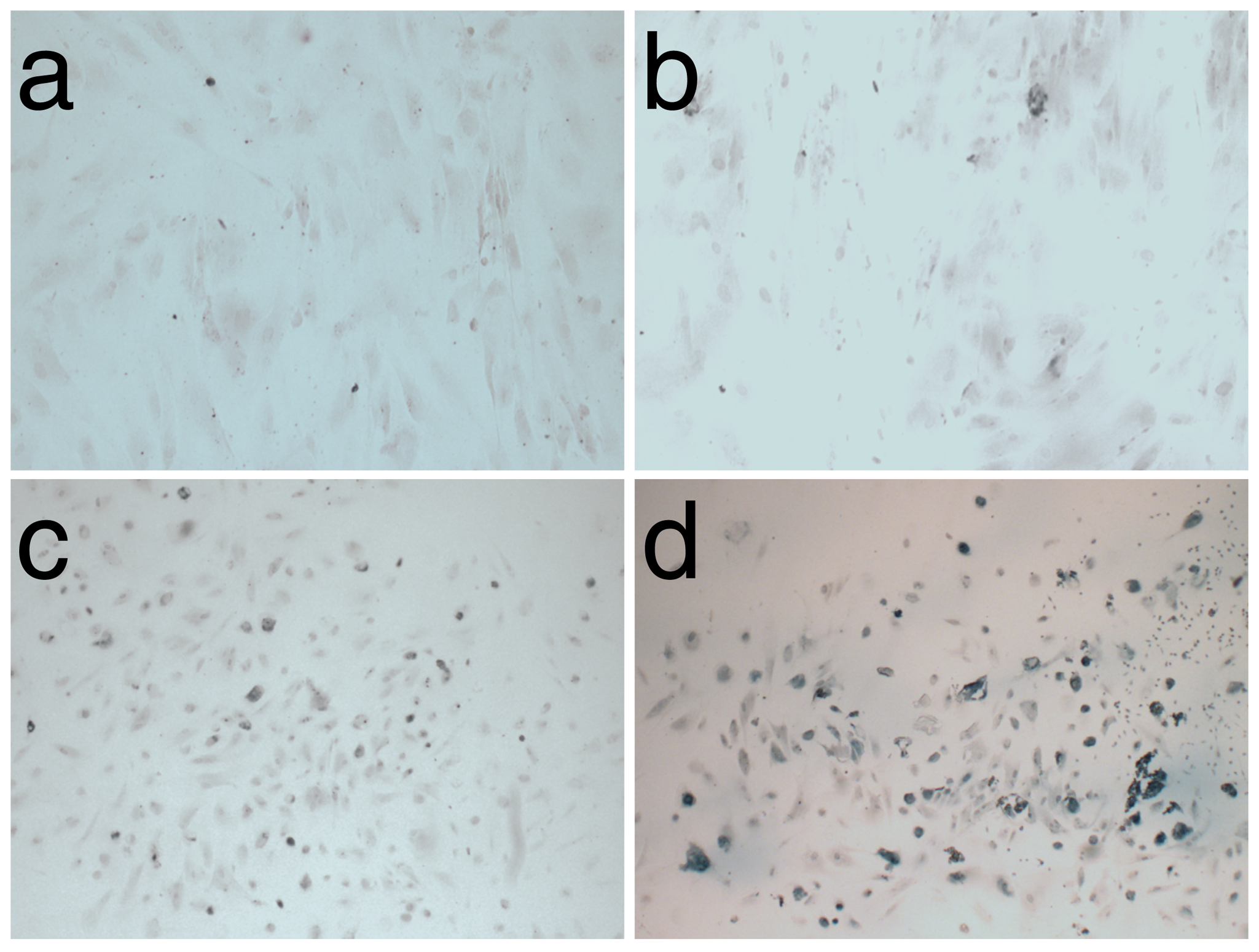

Supplement: S1 Fig — Naïve human UCMSC (a) were transduced by 100 MOI Ad-LacZ (b) 50 MOI (c) and 100 MOI (d) Ad-ADRP. Seven days after transduction, cells were stained by Sudan-black. Human UCMSC transduced with Ad-LacZ showed a negligible amount of oil droplets. In contrast, Ad-ADRP transduction induced a large amount of lipid accumulation in human UCMSC. (TIFF) [file pone.0123756.s001.tiff]
